# RNA-seq reveals the gene expression in patterns in *Populus × euramericana* 'Neva' plantation under different precision water and fertilizer-intensive management

**DOI:** 10.1186/s12870-024-05427-4

**Published:** 2024-08-09

**Authors:** Zhou Wang, Weixi Zhang, Changjun Ding, Yongxiu Xia, Zhengsai Yuan, Jiangtao Guo, Jinjin Yu, Bingyu Zhang, Xiaohua Su

**Affiliations:** 1 State Key Laboratory of Tree Genetics and Breeding, Research Institute of Forestry, Chinese Academy of Forestry, Beijing, 100091 China; 2 Key Laboratory of Tree Breeding and Cultivation of State Forestry Administration, Research Institute of Forestry, Chinese Academy of Forestry, Beijing, 100091 China; 3 Experimental Center of Forestry in North China, National Permanent Scientific Research Base for Warm Temperate Zone Forestry of Jiulong Mountain in Beijing, Chinese Academy of Forestry, Beijing, 100023 P.R. China; 4 Heibei Agricultural University, Baoding, 071001 P.R. China; 5 Co-Innovation Center for Sustainable Forestry in Southern China, Nanjing Forestry University, Nanjing, 210037 China

**Keywords:** *Populus × euramericana*, Precision water and fertilizer-intensive management, Transcriptome, Nitrogen metabolism, Photosynthesis

## Abstract

**Background:**

*Populus* spp. is a crucial fast-growing and productive tree species extensively cultivated in the mid-latitude plains of the world. However, the impact of intensive cultivation management on gene expression in plantation remains largely unexplored.

**Results:**

Precision water and fertilizer-intensive management substantially increased key enzyme activities of nitrogen transport, assimilation, and photosynthesis (1.12–2.63 times than CK) in *Populus × euramericana* 'Neva' plantation. Meanwhile, this management approach had a significant regulatory effect on the gene expression of poplar plantations. 1554 differential expression genes (DEGs)were identified in drip irrigation (ND) compared with conventional irrigation. Relative to ND, 2761–4116 DEGs, predominantly up-regulated, were identified under three drip fertilization combinations, among which 202 DEGs were mainly regulated by fertilization. Moreover, drip irrigation reduced the expression of cell wall synthesis-related genes to reduce unnecessary water transport. Precision drip and fertilizer-intensive management promotes the synergistic regulation of carbon and nitrogen metabolism and up-regulates the expression of major genes in nitrogen transport and assimilation processes (5 DEGs), photosynthesis (15 DEGs), and plant hormone signal transduction (11 DEGs). The incorporation of trace elements further enhanced the up-regulation of secondary metabolic process genes. In addition, the co-expression network identified nine hub genes regulated by precision water and fertilizer-intensive management, suggesting a pivotal role in regulating the growth of poplar.

**Conclusion:**

Precision water and fertilizer-intensive management demonstrated the ability to regulate the expression of key genes and transcription factor genes involved in carbon and nitrogen metabolism pathways, plant hormone signal transduction, and enhance the activity of key enzymes involved in related processes. This regulation facilitated nitrogen absorption and utilization, and photosynthetic abilities such as light capture, light transport, and electron transport, which faintly synergistically regulate the growth of poplar plantations. These results provide a reference for proposing highly efficient precision intensive management to optimize the expression of target genes.

**Supplementary Information:**

The online version contains supplementary material available at 10.1186/s12870-024-05427-4.

## Background

*Populus* spp. stands as one of the foremost fast-growing and prolific tree species, commanding the largest intensive cultivation area in the mid-latitude plains worldwide [1]. Poplar is the most widely planted plantation species in China, with a planting area ranking first in the world. However, the potential production of poplar has not been fully exploited in China, which is much lower than at the international level. One of the key reasons for low production potential is the inefficient intensive management [2].

Water and nutrients complement and interact with each other throughout the entire process of plant growth and development [3, 4], and irrigation and fertilization can cover the shortage of water and nutrients during the life cycle [5]. However, water deficit limits nitrogen uptake whereas over-supplement of water may cause nutrient losses through leaching, thus it is necessary to develop effective application regimes of water and fertilization [6]. Drip fertigation, a new technology combining drip irrigation and fertilization, ensures the simultaneous supply of water and fertilizers [7] and has now been widely applied in intensive agriculture, horticulture, and fruit planting in most developed or agricultural countries, to increase the production of them, as well as water and nutrient utilization efficiency [8, 9]. With the growing awareness of sustainable development, drip fertigation has gained widespread adoption in intensively managed agroforestry, including plantation [3, 8, 10, 11]. Yan et al. [12, 13] have applied drip fertigation in an intensively managed poplar (*Populus × euramericana* 'Guariento') plantation for several years, resulting in significant increases in both biomass and soil organic carbon content compared to conventional management. Specifically, over the three experimental years, the biomass increased by 4.3–52.2%, carbon storage increased by 76%, and the soil organic carbon content exhibited an annual increase of 12–21%. This suggests that applied drip fertigation in intensive management could significantly promote the productivity and increased carbon storage of poplar plantation, as well as the effectiveness of nitrogen and water in the surface soil. Mi et al. [14, 15] analyzed the rules of uptake, consumption, and transport of water and nutrients in the soil, growth plasticity, as well and root spatial distribution of poplar plantation in sandy areas formulated a precision drip fertigation regimes, which could effectively promote the growth of diameter at breast height (DBH), height, and timber volume of poplar plantation, and then increase the productivity. Zhang et al. [16] demonstrated that long-term irrigation significantly increased the total branch xylem cross-sectional area in various canopy layers. This improvement enhanced the overall water-conducting area of the branches within the canopy and then enhanced the growth of *Populus tomentosa* Carr. effectively. Du et al. [17] indicated that the total biomass of *Populus deltoides* 'Danhong' and *Populus simonii* 'Tongliao1' increased 1.69 and 1.10 times, respectively, after fertilization compared with non-fertilization. However, the above studies were mainly concerned with the phenotypic level such as growth, physiology, and biochemistry, there is no study on the effects of cultivation managed on gene expression of plantation.

High-throughput sequencing technology can effectively investigate genome‑wide gene expression of plants, which benefits the explanation of the regulatory effects of different cultivation measures at the omics level, such as the transcriptome, proteome, and metabolome in the field. Based on this, it can clarify the response of gene expression to specific environmental conditions comprehensively, systematically, and realistically, and contribute to proposing a reasonable intensive management with real-time growth regulation technology systems, to achieve the optimal expression of target genes. In recent years, there have been studies reporting on the applications of RNA-sequencing (RNA-seq) technology in intensive agriculture, showing notable progress. Chen et al. [18] analyzed the effects of trace irrigation at different depths on cotton (*Gossypium hirsutum* L.) yield and plant responses in the field, and found that drip irrigation at 30 cm underground can significantly increase cotton yield, which was suitable for cotton irrigation in China’s Inner Mongolia. Nonetheless, drip irrigation at a depth of 50 cm led to a reduced cotton yield and differential expression of transcription factors (including *bZIP*,* WARK*,* Myb*, and *NAC*) in response to drought stress. This implies that irrigation at a greater depth influenced cotton yield by inducing drought stress. Zhang et al. [19] found that stable soil water content (SW) conditions not only increased maize (*Zea mays* L.) growth and yield significantly but also highly up-regulated expression of lots of DEGs in the photosynthesis (including *PsbE*, *PsbF*, *PsbA*, *PsbD*, etc.) and oxidative phosphorylation pathway (including *atpE*, *atpB*,* ndhE*, *ndhG*, etc.), compared with a soil moisture content of dry and wet alternation conditions, which indicates that the physiological mechanism of SW to increase maize yield may be the enhancement of photosynthetic capacity and energy metabolism. Ou et al. [20] studied the effects of different nitrogen fertilizer treatments on transcriptome variations of *Panax notoginseng* roots and found that ammonium and nitrate fertilizers are simultaneously used could increase the *P. notoginseng* root yield by promoting the TCA cycle, which activated by up-regulation of ACLA-3 and several key metabolites in this cycle. Fu et al. [21] researched how rice (*Oryza sativa* L.) responded to the mixed provision of ammonium- and nitrate-nitrogen(MPAN)and found that the amount and rate of nutrient (N, P, and K) uptake and their translocation in rice were highly enhanced under 75:25 MPAN with 25% of NO_3_^−^-N and shoot biomass was also increased significantly under 75:25 MPAN. Ultimately, 476 DEGs (288 up-regulated and 179 down-regulated) associated with nitrogen metabolism, carbon fixation in photosynthetic organisms, photosynthesis, starch and sucrose metabolism, and zeatin biosynthesis were identified. These genes play a crucial role in enhancing nutrient uptake, translocation, and seedling growth. However, so far, there has been only one study on the different cultivation measure’s effects on gene expression of plantation forestry. That is the gene expression of plantation poplar (*Populus* × *euramericana*) regulated by different planting densities, first published by our research group. This study showed that there were significant changes in the expression of metabolism-related and stimulus-related genes in response to planting density. And number of genes related to plant light responses, photosynthesis, and carbon and nitrogen metabolism were observed, displaying upregulation under high-density [22].

Here, an 11-year-old *Populus × euramericana* 'Neva' plantation in the sandy area of the North China Plain was used as the research object, we analyzed the whole-genome expression patterns of leaves under different combine of water and fertilizer cultivation for several consecutive months by RNA-seq. Together with growth and the activity of enzymes related to carbon and nitrogen metabolism, to reveal the effects of precision water and fertilizer-intensive management on gene expression to identify the core related genes. This will provide a reference basis for the proposal of highly efficient precision intensive management to achieve the optimal expression of the target genes.

## Materials and methods

### Site description and sampling

The experiment site is located in Yufa Town Forestry farm, Daxing District, Beijing, China, which belongs to the warm-temperate semi-humid continental monsoon climate. The region experiences an average annual sunshine duration of 2620.4 h, with an average annual temperature of 11.6 °C. Winters are characterized by an average temperature of -2.3 °C, while summers see an average temperature of 25.1 °C. Annual precipitation averages 552.9 mm, and evaporation ranges between 1800 and 2000 mm annually. The frost-free period spans 180–200 days each year. The soil in the experiment site is sandy soil alluvial from the old course of the Yongding River. The soil profile investigation shows that there is no obvious humus layer from the surface down to 1.2 m, with uniform texture of fine sand, poor comprehensive soil fertility, and low productivity.

Eleven-year-old *Populus × euramericana* 'Neva' were used as experimental materials. The plantation had a row spacing of 3 m × 5 m and was growing normally without pests or diseases. Two irrigation methods, three fertilization conditions, and two fertilization gradients were set up in the experiment. There are a total of 7 types of water and fertilizer treatments: no fertilization conventional irrigation (CK), no fertilization drip irrigation (ND), water and fertilizer coupling: controlled release fertilizer drip irrigation (CD, drip -fertilizer combine), water-soluble fertilizer drip irrigation (M1D, M2D, drip-fertilizer integration), formula fertilizer drip irrigation (F1D, F2D, drip-fertilizer with trace element integration). The fertilization rates under CD, M1D, M2D, F1D and F2D treatments were approximately 3:1:2 for the ratio of N, P, and K(The fertilizer formulas employed in the study are detailed in Table S1). Conventional irrigation is implemented three times annually, whereas drip irrigation is administered a total of 15 times throughout the year. Different irrigation methods ensure consistent soil moisture content at the same soil depth. Combining water-soluble fertilizer and formula fertilizer with drip irrigation, opening the fertilization system, and fertilizing with water; Controlled release fertilizer will be applied in one go in May.

Before sampling, 5 standard plants were selected for positioning in each treatment group, and side trees were removed. In mid-May (before fertilization), mid-July, mid-August, and mid-September, 3–4 pieces of functional leaves of the current year’s branches growing southward in the top 1/3 of the trees on each standard plant were collected, immediately placed in liquid nitrogen for quick-freezing and then stored at -80 °C in a freezer. 5 biological replicates were set up for the determination of physiological indicators, totaling 140 samples; 3 biological replicates were set up for transcriptome sequencing, totaling 84 samples.

### Determination of physiological indicators

The activities of nitrate reductase (NR), glutamate dehydrogenase (GDH), glutamate synthase (GOGAT), ribulose bisphosphate carboxylase/oxygenase (Rubisco), fructose-1,6-bisphosphate aldolase (FBA) and the contents of soluble sugar (SS) and chlorophyll (Chl) in poplar leaves were determined using a micromethod. For details on the assay method, refer to the corresponding product instructions on the Solarbio website (https://www.solarbio.com/index.php).

### RNA extraction, cDNA library construction, and RNA sequencing

Total RNA was extracted using TRIzol Reagent (Invitrogen, USA) according to the manufacturer’s protocol. RNA purity was checked using the kaiaoK5500^®^Spectrophotometer (Kaiao, Beijing, China), RNA integrity and concentration were assessed using the RNA Nano 6000 Assay Kit of the Bioanalyzer 2100 system (Agilent Technologies, CA, USA), transcriptome sequencing libraries were constructed after the samples were tested and approved. Transcriptome sequencing libraries were constructed according to the instructions of the NEBNext Ultra RNA Library Prep Kit for Illumina (#E7530L, NEB, USA). Sequencing was performed with the Illumina NovaSeq 6000 (Illumina, USA) platform with the sequencing strategy PE150. Raw reads from Illumina platform flat sequencing are processed to obtain high-quality sequences (Clean Reads) by removing low-quality sequences, decontaminating junctions, etc., and calculating Q20, Q30, and GC content of the Clean Data. The Eukaryotic Transcriptome (with reference genome) Sequencing was realized by Annoroad Gene Technology Co., Ltd (Beijing, China).

### RNA-Seq data processing and analysis

Bowtie2 v2.2.3 was used for building the genome index and then Clean Data was compared to the *Populus deltoides* genome database (https://phytozome-next.jgi.doe.gov/info/PdeltoidesWV94_v2_1) by HISAT2 v2.1.0 [23]. Reads Count for each gene in each sample was counted by HTSeq v0.6.0, and FPKM (Fragments Per Kilobase Millon Mapped Reads) was then calculated to estimate the expression level of genes in each sample [24]. Genes with *q* ≤ 0.05 and |log2_ratio| ≥ 1 are identified as DEGs [25]. Gene Ontology (GO) and KEGG (Kyoto Encyclopedia of Genes and Genomes) enrichment analysis of DEGs was performed using the OmicShare tools (https://www.omicshare.com/tools) and Weighted correlation network analysis(WGCNA) analysis using the OE Cloud tool (https://cloud.oebiotech.com).

### Quantitative real-time PCR verification

To verify the reliability of the RNA-seq results, 11 randomly selected genes were analyzed at the gene expression level. Quantitative real-time PCR (qRT-PCR) was performed using a Light Cycler 480 Instrument II system (Roche, Switzerland) and analyzed with SYBR Premix Ex Taq II (Takara) using the following parameters: 95 °C for 30s, 40 cycles of 95 °C for 5s and 60 °C for 30s, followed by 95 °C for 5s, 60 °C for 1 min, and 95 °C with continuous acquisition mode at per 5 °C, with a final extension at 50 °C for 30s. The assay was repeated three times per sample after mixing. Actin (OX637672.1) was used as an internal reference gene. Gene-specific primers were designed (Table S2). The relative expression of genes was calculated using the 2^−∆∆Ct^ [26].

## Results

### Impact of precision water and fertilizer-intensive management on physiological and biochemical indicators of poplar

**Fig. 1:**
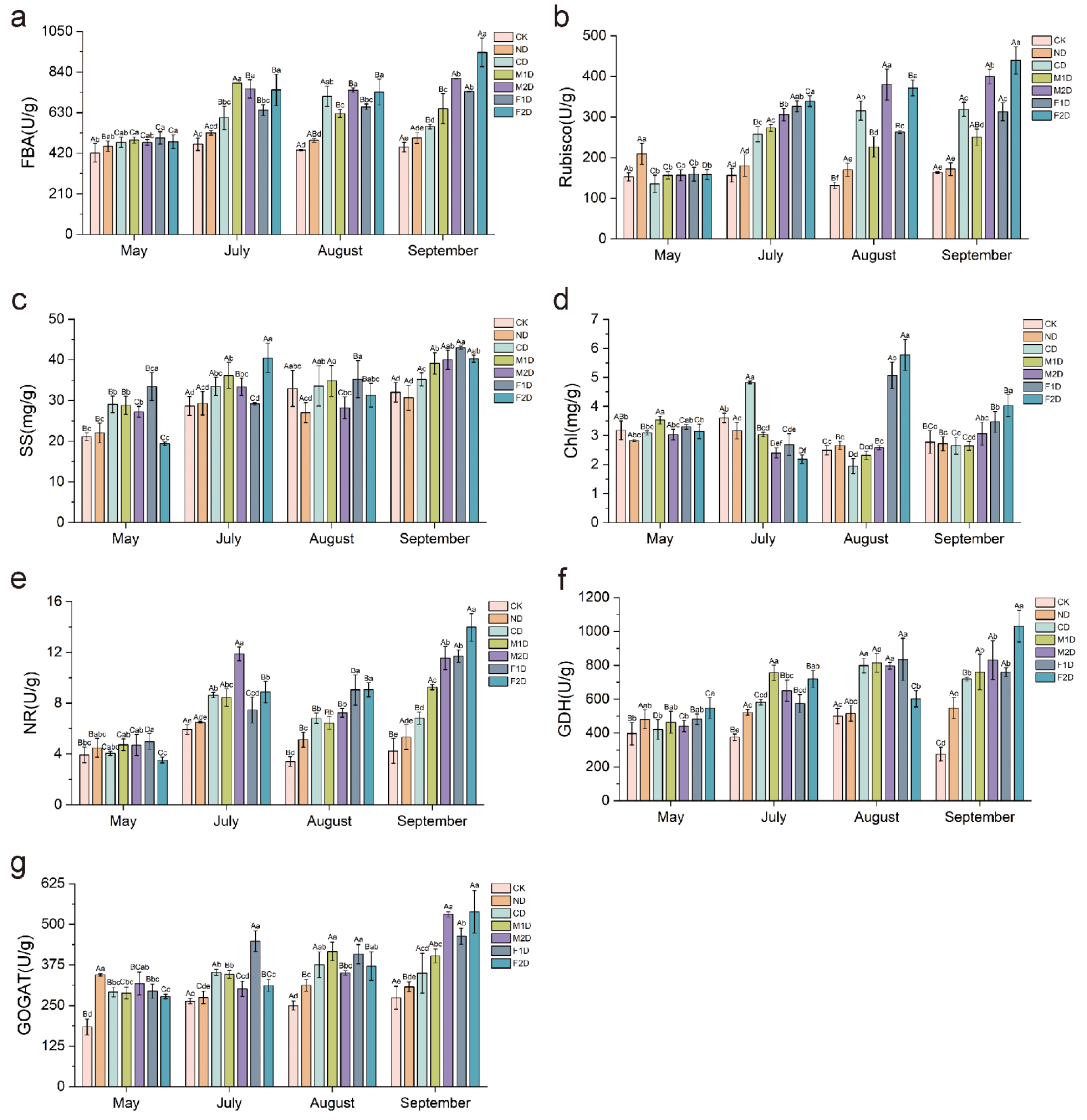
Measurement of physiological and biochemical indicators of poplar leaves under different water and fertilizer treatments and months. **a** FBA activity. **b** Rubisco activity. **c** SS content. **d** Chl content. **e** NR activity. **f** GDH activity. **g** GOGAT activity. The error bar represents the standard deviation (*n* = 3). Capital letters indicate significance between months for the same treatment, lowercase letters indicate significance between treatments for the same month, and different letters indicate significant differences (*P* < 0.05)

To investigate the effects of precision water and fertilizer-intensive management on the physiological and biochemical indicators of poplar, the activities of fructose-1,6-bisphosphate aldolase (FBA), glutamate dehydrogenase (GDH), glutamate synthase (GOGAT), nitrate reductase (NR), ribulose bisphosphate carboxylase/oxygenase (Rubisco), as well as the content of soluble sugar (SS) and chlorophyll (Chl) in poplar leaves were measured under different water and fertilizer treatments (CK, ND, CD, M1D, M2D, F1D, and F2D). The findings revealed that the alterations in FBA, GDH, GOGAT, NR, Rubisco activity, and SS content exhibited similar patterns under distinct water and fertilizer treatments (Fig. 1). Compared with CK, the activity of GOGAT, NR, and SS content under ND increased by 1.04, 1.10, and 1.02 times in July, respectively. In August, the GDH activity under ND was 1.03 times that of CK. In September, the activity of Rubisco under ND increased by 1.05 times. These above results indicated that although various physiological and biochemical indicators under ND increased compared to CK, the differences were not significant. Compared with ND, the activities of GDH, GOGAT, NR, Rubisco, and SS content increased by 16.95 – 46.09% under CD treatment in August. M1D increased the activities of FBA, GDH, GOGAT, NR, Rubisco, and SS content by 18.99 – 42.55% in July, August, and September. In September, the activities of FBA, GDH, GOGAT, NR, Rubisco, and SS content under M2D and F1D treatments were approximately 1.30–2.32 times and 1.39–2.20 times that of ND, respectively. The activities of FBA, GDH, GOGAT, NR, Rubisco, and SS content under F2D treatment increased by 11.76 – 46.94% and 23.81 – 61.95% in July and September, respectively. These findings suggest a significant increase in all the aforementioned physiological and biochemical indicators of poplars under water-fertilizer coupling (CD, M1D, M2D, F1D, and F2D) compared to ND. Comparing the differences between the same treatment in different months, it was found that various physiological and biochemical indicators after water and fertilizer treatment (July, August, and September) significantly increased compared to those before treatment (May). Compared with May, the activity of FBA in ND treatment increased by 1.1 times in July, August, and September; the activity of GDH increased by 1.1 times and 1.68 times under ND and M1D, respectively; the activity of GOGAT increased by about 18.76% and 32.92% in CD and F1D; the activity of Rubisco increased by 1.45–1.74 times and 2.14–2.78 times under M1D and F2D treatments, respectively; the content of SS increased by about 27.52%, 15.70%, and 24.08% under ND, CD, and M1D, respectively. Additionally, there was no discernible change pattern in the chlorophyll (Chl) content, but in August and September, the Chl content significantly increased under F1D and F2D, rising by 21.61–53.98% compared with ND. In summary, we believe that different irrigation methods have a relatively small impact on the physiological and biochemical indicators of poplar, but the coupling of water and fertilizer significantly increases physiological and biochemical indicators such as photosynthesis and nitrogen metabolism-related enzyme activity compared to drip irrigation, indicating that nutrient addition may be the main factor affecting the physiological and biochemical characteristics of poplar; The Chl content showed a significant increase in drip fertilization with trace element integration, indicating that the addition of trace elements may promote the increase of Chl content.

## Global analysis of RNA-Seq

To clarify the gene expression pattern of poplar plantation leaves under precision water and fertilizer management, RNA-seq analysis was performed, and a total of about 521.5 G clean data were obtained, with an average of about 6 G clean reads per sample, the percentage of Q30 was above 88%, and the GC contents were all around 45%, and the clean reads were compared to the PdeltoidesWV94_v2_1 reference genome, the matching rate per sample was above 82%, with the unique matching rate above 70% (Table S4). The FPKM method was employed for the quantitative estimation of gene expression values, revealing a total of 29,763 genes expressed in at least one sample (Table S5). The Pearson correlation coefficient (Fig. S1) was employed to assess the correlation between the biological replicates of each sample. The Pearson correlation coefficient between the biological repeats ranged from 0.90 to 0.99, indicating the high reliability of the RNA-seq data.

### Differentially expressed genes and patterns under precision water and fertilizer-intensive management

**Fig. 2:**
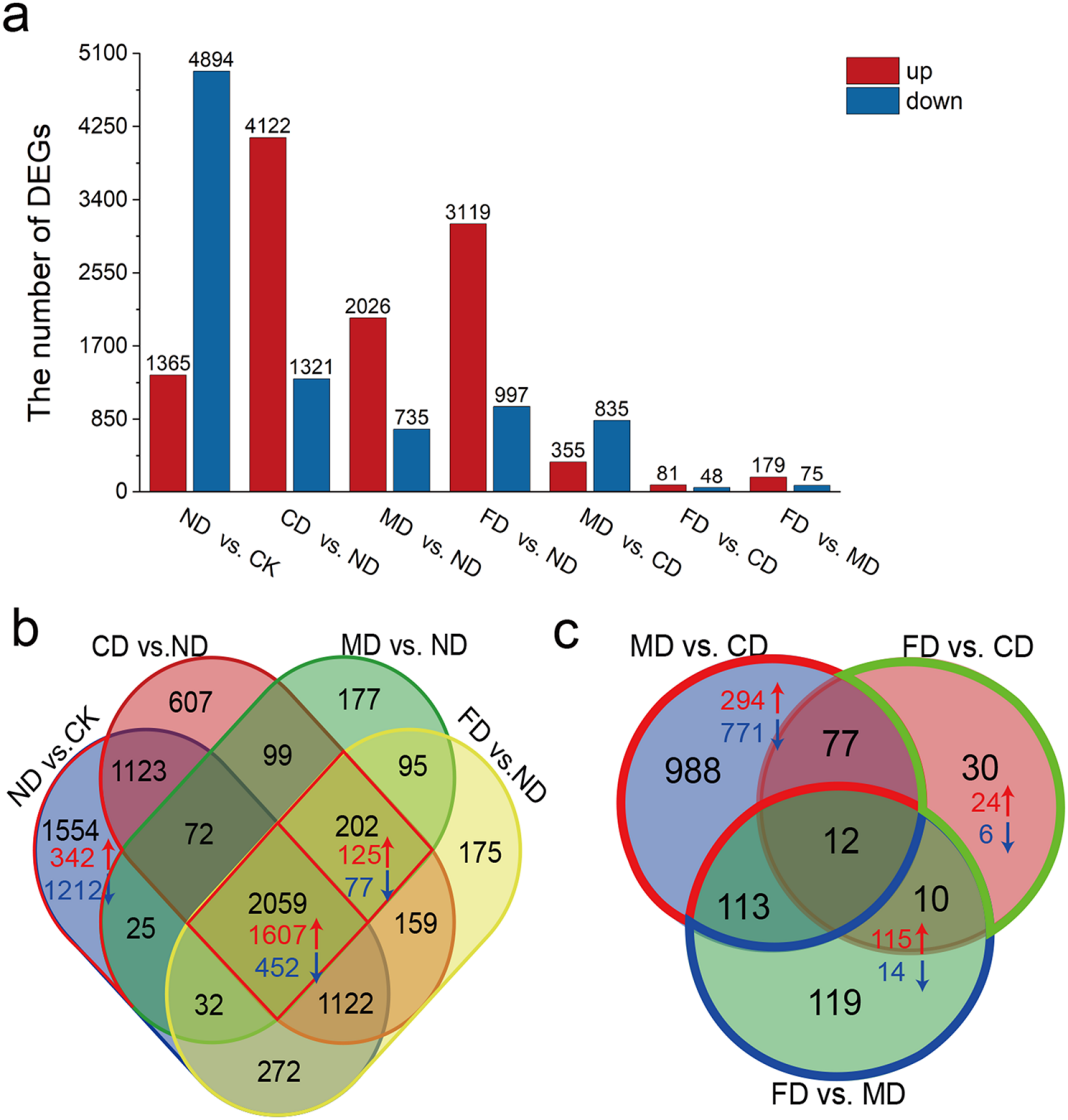
The number of DEGs and Venn diagram in different comparison groups in September. **a** The number of up- and down-regulated DEGs in different comparison groups in September. **b** Venn diagram of DEGs between ND vs. CK, CD vs. ND, MD vs. ND, and FD vs. ND. **c** Venn diagram of DEGs between MD vs. CD, FD vs. MD, and FD vs. CD. The red arrow indicates up-regulation of gene expression, while the blue arrow indicates down-regulation of gene expression

Using | log2Fold change | ≥ 1, q < 0.05 as the screening threshold, perform DEGs analysis (Table 1). The number of DEGs was significantly higher in September (2344–5943) than in May, July, and August (1–222), indicating that genes may strongly respond to precision water and fertilizer-intensive management in September. The number of DEGs for the same fertilizer under different fertilization gradients (M1D and M2D, F1D and F2D) is 4–16, indicating that the study set fertilization gradients to regulate gene expression weakly. Therefore, we only conducted differential expression analysis on the genes of CK, ND, CD, MD (non-gradient water-soluble fertilizer), and FD (non-gradient formula fertilizer) treatments (Fig. 2a). A total of 6259 DEGs (1365 up-regulated and 4894 down-regulated) were identified in ND vs. CK, suggesting that drip irrigation may negatively regulate the expression of genes. In CD vs. ND, MD vs. ND, and FD vs. ND, there were 5443, 2761, and 4116 DEGs, respectively. The trends of gene expression were consistent across the three different water and fertilization treatments, with the DEGs being predominantly up-regulated in expression, compared to drip irrigation alone. In MD vs. CD (1190 DEGs; 355 up-regulated, 835 down-regulated), FD vs. CD (129 DEGs, 81 up-regulated, 48 down-regulated), and FD vs. MD (254 DEGs, 179 up-regulated, 75 down-regulated), the changes in gene expression were also observed.

To analyze the co-expressed and specific genes under different treatments, a Venn analysis was performed. There were 1554 specifically DEGs in ND vs. CK with 342 up-regulated DEGs and 1212 down-regulated DEGs, which might be mainly down-regulated by drip irrigation. 2059 DEGs were co-expressed in ND vs. CK, CD vs. ND, MD vs. ND, and FD vs. ND (1607 up-regulated, 452 down-regulated under water-fertilizer coupling) four comparison groups, which might be mainly up-regulated by water and fertilizer treatment. 202 DEGs were co-expressed in CD vs. ND, MD vs. ND, and FD vs. ND (125 up-regulated, 77 down-regulated), which suggests these genes may be positively regulated by nutrients (Fig. 2b). In addition, we found that some DEGs were affected by different water and fertilizer treatment methods (Fig. 2c), among which 1065 DEGs (294 up-regulated, 771 down-regulated in MD vs. CD) were regulated by drip-fertilizer integration, 129 DEGs (115 up-regulated, 14 down-regulated in FD vs. MD) might be regulated by trace elements.

**Table 1:** Number of DEGs under precision water and fertilizer-intensive management

	May	July	August	September
ND vs. CK	938	379	219	5943
CD vs. ND	1194	237	222	5011
M1D vs. ND	65	156	94	2344
M2D vs. ND	2	636	55	2405
F1D vs. ND	130	73	1	3472
F2D vs. ND	128	108	11	3548
F2D vs. F1D	26	5	122	4
M2D vs. M1D	0	116	13	16
F1D vs. CD	177	2	71	191
F1D vs. M1D	14	40	193	143
F2D vs. M2D	0	0	0	247
M1D vs. CD	195	42	87	1314

### Function annotation of the DEGs

**Fig. 3:**
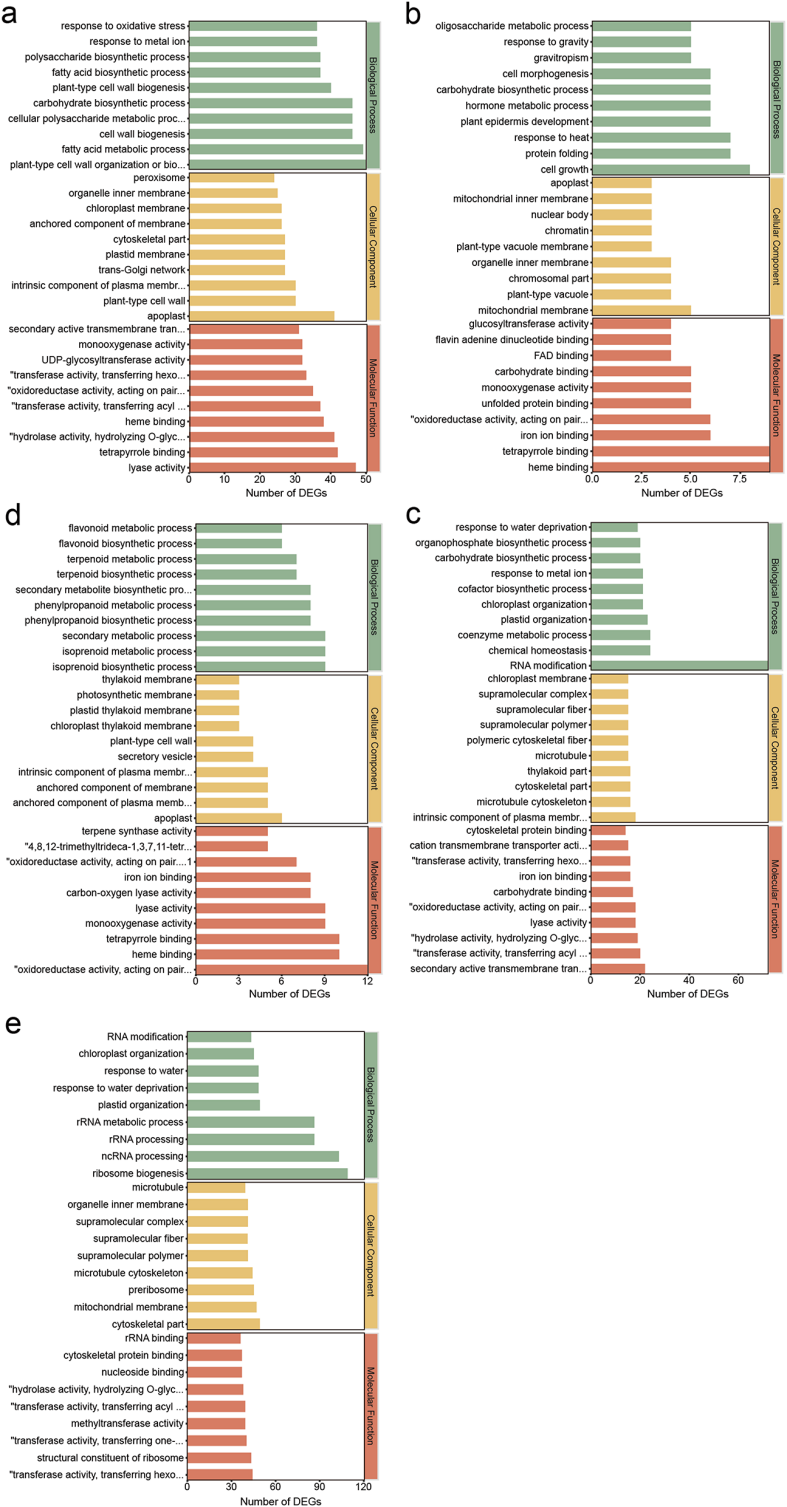
GO enrichment analysis of DEGs regulated by different treatments. Bars of different colors indicate different Categories. **a** The enriched GO terms of genes regulated by drip irrigation. **b** The enriched GO terms of genes regulated by fertilizers. **c** The enriched GO terms of genes regulated by drip-fertilizer integration. **d** The enriched GO terms of genes regulated by trace elements. **e** The enriched GO terms of genes regulated by water and fertilizer treatment

To categorize the functional roles of DEGs, a GO analysis was conducted, classifying the DEGs based on their involvement in biological processes (BP), cellular components (CC), and molecular functions (MF). Genes in the biological process were enriched in the richest variety and number, and there were some unique GO terms (Fig. 3). DEGs regulated by drip irrigation were highly enriched in the plant cell wall and in the GO term occurring in the plant cell wall; in terms of MF, significant enrichment was observed in functions such as various enzyme activity and "FMN binding" (Fig. 3a). Fertilizer-regulated DEGs were mainly enriched in BP such as "cell growth", "protein folding", and "response to heat"; in cellular components, they were significantly enriched in CC such as "amyloplasts" and "mitochondrial intermembrane space"; and in MF, more DEGs were enriched in "heme binding" and "tetrapyrrole binding" (Fig. 3b). DEGs regulated under drip-fertilizer integration were notably enriched in various BPs, including "RNA modification" and "chemical homeostasis". In terms of CC, the primary enrichment was observed in "chloroplast nucleoid" and "plastid nucleoid". Regarding MF, the DEGs showed significant enrichment in functions such as "inorganic anion exchanger activity" and "glucose transmembrane transporter activity" (Fig. 3c). The GO terms that were significantly enriched in DEGs regulated by trace elements included various secondary metabolic processes, in terms of CC, they were mainly enriched in the "apoplast" (Fig. 3d). DEGs regulated by water and fertilizer treatment were significantly enriched in BP such as "ribosome biogenesis" and "rRNA processing", and were also enriched in components such as "mitochondrion" and "cytoskeleton" and molecular function such as "mitochondrial ribosome binding" and "snoRNA binding" (Fig. 3e).

**Fig. 4:**
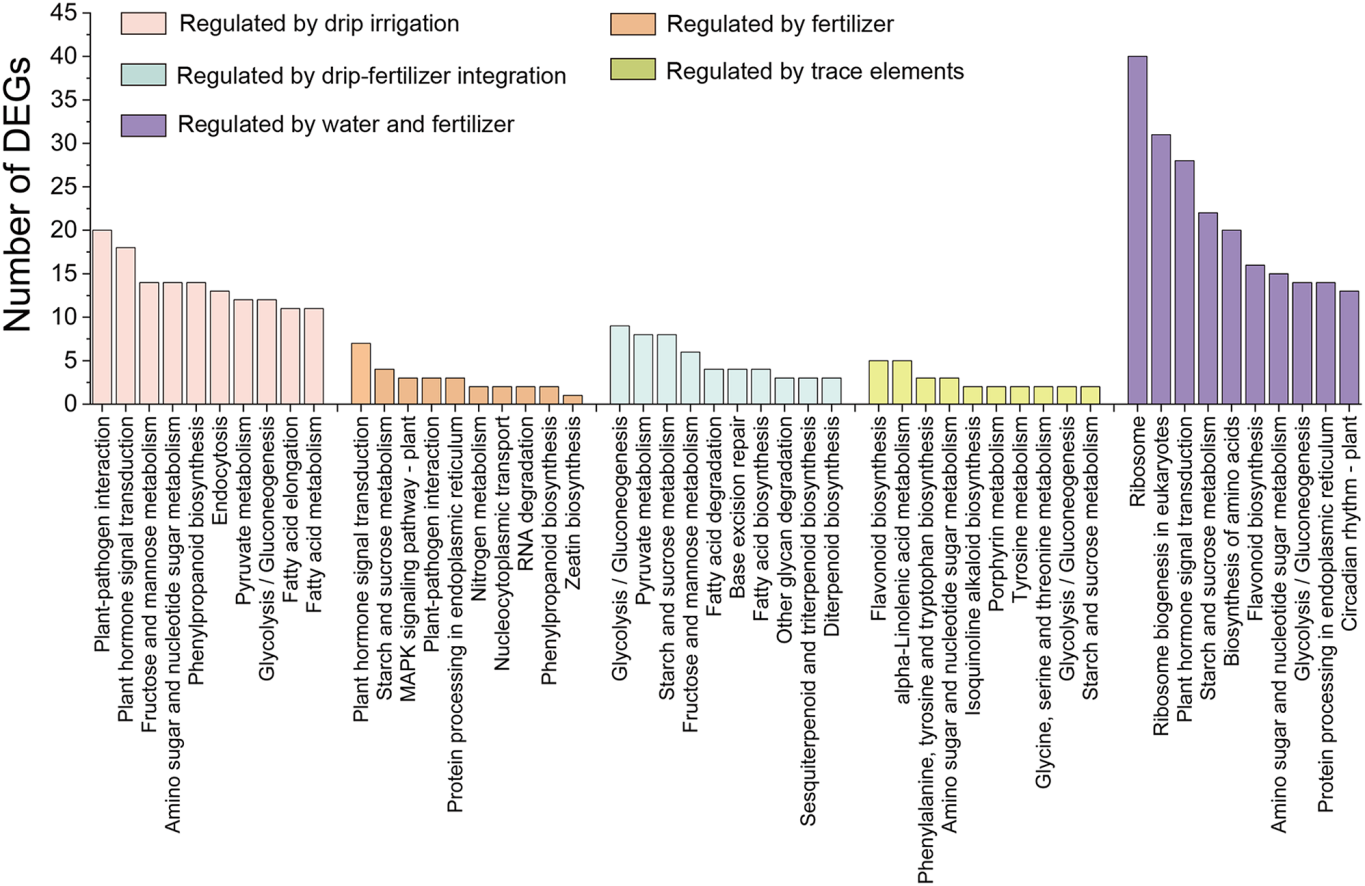
Top 10 pathways of KEGG enrichment analysis of DEGs regulated by different treatments. The bar with different colors represents the DEGs regulated by different water and fertilization treatments

KEGG pathway analysis was used to further understand the biological functions of DEGs identified under precision water and fertilizer-intensive management (Fig. 4). The results indicated that common KEGG pathways affected by different water and fertilizer treatments included carbohydrate metabolic processes such as "fructose and mannose metabolism" and "starch and sucrose metabolism". In addition, there are some specific KEGG pathways, DEGs regulated by nutrients are highly enriched in "plant hormone transduction signaling" and "nitrogen metabolism"; DEGs regulated by trace elements are more enriched in "flavonoid biosynthesis" and "α-linolenic acid metabolism" and other pathways; DEGs significantly enriched in the pathways regulated by water and fertilizer treatment are mainly involved in the processing of genetic information, secondary metabolites, and nucleotide metabolism, including "Ribosome biogenesis in eukaryotes", "Flavonoid biosynthesis", etc.

### DEGs related to photosynthesis

**Fig. 5:**
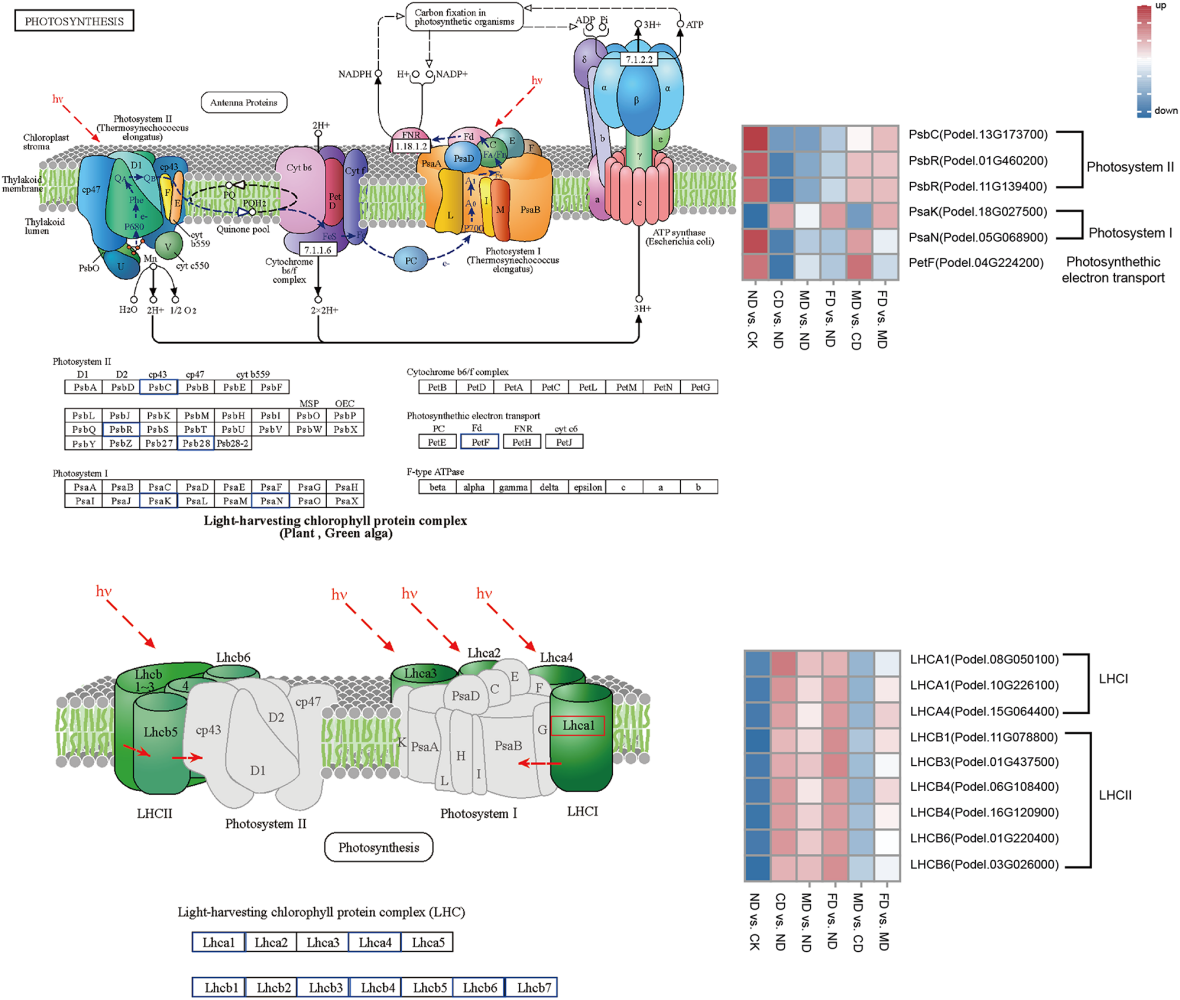
Expression of DEGs related to photosynthesis. Citation guidelines: www.kegg.jp/kegg/ kegg1. html. The rectangles of different colors indicate the up-/down-regulated expression of genes, red indicates up-regulation, blue indicates down-regulation and the depth of the color indicates the level of up-and down-regulation, the darker the color, the more significant the up-/down-regulation, as below

Photosynthesis, one of the fundamental biological processes in higher plants, plays a crucial role in converting the pure energy of light into the biochemical energy essential for life through a series of reactions [27]. We found 15 DEGs related to photosynthesis (Fig. 5), the important constitutive proteins in photosystem II PsbR (photosystem II 10 kDa protein, Podel.01G460200 and Podel.11G139400) and PsbC (photosystem II CP43 chlorophyll apoprotein, the Podel.13G173700), PsaN (photosystem I subunit PsaN, Podel.05G068900), an important constituent protein, and PetF (ferredoxin, Podel.05G068900), photosynthetic electron transport (Podel.04G224200) genes had similar expression trends, and they were both up-regulated in ND vs. CK and MD vs. CD and down-regulated in CD vs. ND, MD vs. ND, and FD vs. ND. On the other hand, the expression of PsaK (photosystem I subunit X, Podel.18G027500) in photosystem I was up-regulated in CD vs. ND, MD vs. ND, and FD vs. ND and FD vs. MD. In addition we found some genes of light-harvesting antenna complex (LHC), LHCA1 (Chlorophyll a-b binding protein 6, Podel.08G050100 and Podel.10G226100) in LHC I, LHCA4 (Chlorophyll a-b binding protein 4, Podel.15G064400), and LHCB1 (Chlorophyll a-b binding protein 40, Podel.11G078800), LHCB3 (Chlorophyll a-b binding protein 13, Podel.01G437500), LHCB4 (Chlorophyll a-b binding protein CP29.2, Podel.06G108400 and Podel.16G120900), LHCB6 (Chlorophyll a-b binding protein CP24 10 A. Podel.01G220400 and Podel.03G026000) were all up-regulated in CD vs. ND, MD vs. ND, FD vs. ND, and FD vs. MD, and down-regulated in ND vs. CK and MD vs. CD.

Thus, it is clear that the gene expression of precision water and fertilizer-intensive management has a significant role in regulating photosynthesis. The expression of related genes in the photosystem was up-regulated under drip irrigation and drip-fertilizer integration, so we hypothesized that these two treatments could drive photosynthesis and electron transfer reactions in poplar. Fertilization and addition of trace elements then up-regulated the expression of related genes in photosynthetic antenna proteins, indicating that the addition of fertilizer and trace elements facilitates the capture and utilization of light energy by poplar.

### DEGs related to nitrogen metabolism

**Fig. 6:**
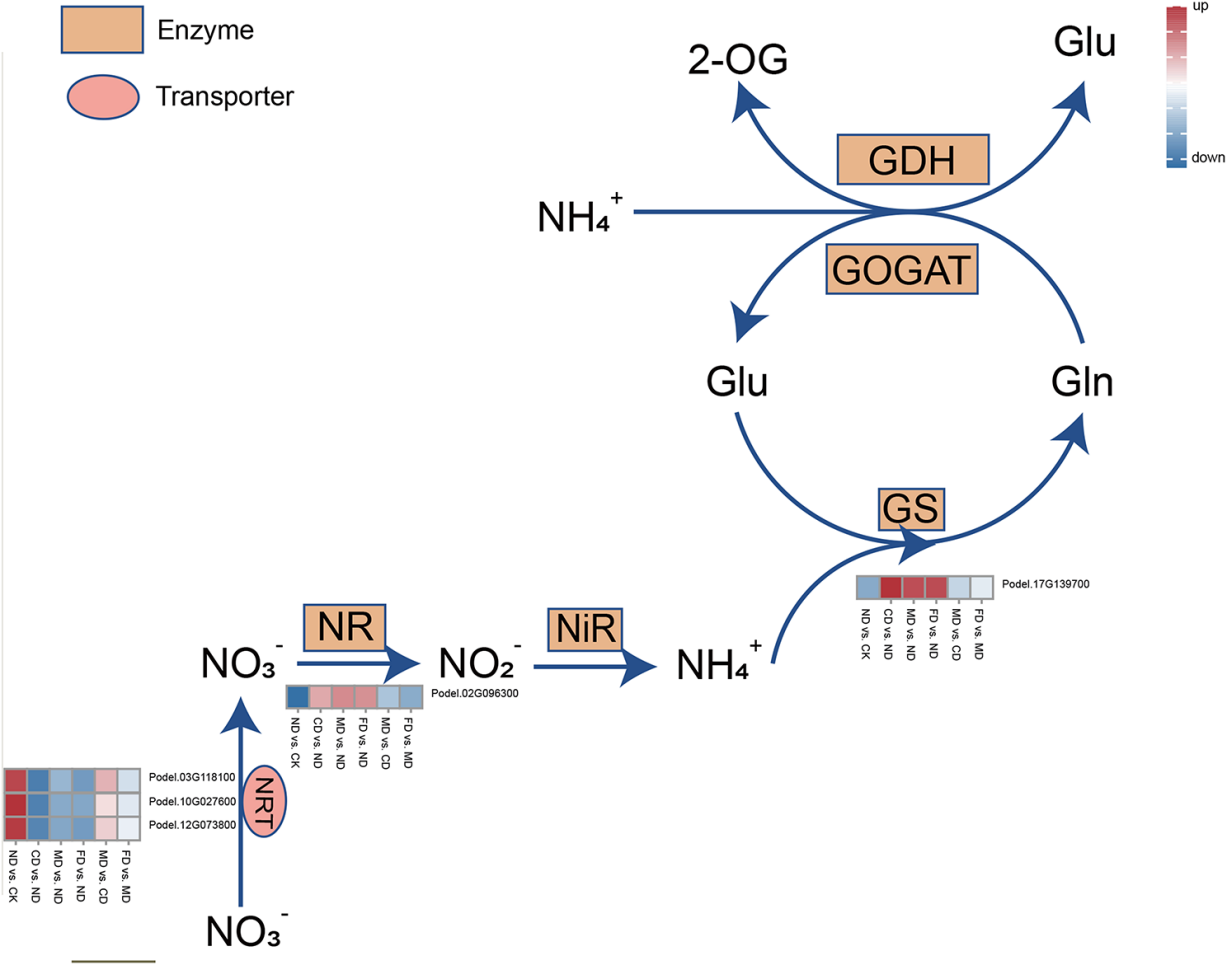
Expression of DEGs related to nitrogen metabolism

Nitrogen metabolism refers to the whole process of uptake, assimilation, and utilization of nitrogen in plants, and is one of the basic metabolic pathways in plants [21]. Based on the functional annotation results, we found that 3 genes encoding NRT (Nitrate transporter, Podel.03G118100, Podel.12G073800 and Podel.14G167600) were significantly differentially expressed in ND vs. CK and CD vs. ND, MD vs. ND and FD vs. ND; the genes encoding NR (Nitrate reductase, Podel.02G096300) and GS (Glutamine synthetase, Podel.17G139700) were significantly differentially expressed in CD vs. ND and MD vs. ND and FD vs. ND. The genes for glutamine synthetase (GS) and nitrate reductase (NR) were found to be significantly differentially expressed in CD vs. ND, MD vs. ND, and FD vs. ND. Based on the significant regulation of these five genes by varying water and fertilizer treatments, we hypothesized that they are important for nitrogen metabolism (Fig. 6). NRTs were up-regulated in ND vs. CK and MD vs. CD expression and down-regulated in CD vs. ND, MD vs. ND, FD vs. ND, and FD vs. MD expression; and GS was up-regulated in CD vs. ND, MD vs. ND, and FD vs. ND expression and down-regulated in ND vs. CK, MD vs. CD, and FD vs. MD expression. It can be seen that the expression of genes related to the nitrogen metabolism process under drip irrigation and water-fertilizer coupling treatments had opposite regulation patterns and affected nitrate transport by regulating the expression of NRT, NR, and GS were significantly up-regulated under water-fertilizer coupling treatment, which indicated that water-fertilizer coupling could effectively promote the process of nitrogen uptake.

### DEGs related to plant hormone signal transduction

**Fig. 7:**
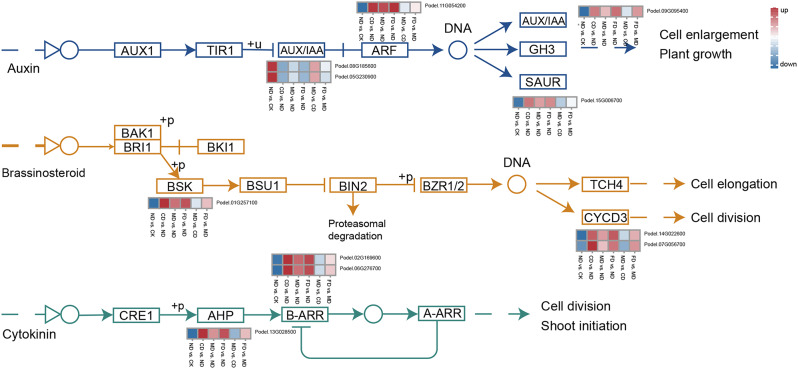
Expression of DEGs related to plant hormone signal transduction

Plants have developed multiple mechanisms and complex signaling networks during their long-term evolutionary process to rapidly sense the external environment and regulate gene expression to adjust their growth and development, as well as to adapt to, resist, and tolerate various biotic and abiotic stresses [28]. Therefore, we analyzed the role of a total of 11 DEGs in plant hormone signal transduction (Fig. 7).

In the auxin signaling pathway, we found that genes encoding ARF (auxin response factor, Podel.11G054200), GH3 (auxin responsive GH3 gene family, Podel.09G095400), and SAUR (SAUR family protein, Podel.15G006700) were down-regulated in ND vs. CK comparison group, up-regulated in CD vs. ND, MD vs. ND, and FD vs. ND comparison groups, but the differential expression between MD vs. CD and FD vs. MD was not significant, indicating that fertilizer addition may be an important factor in enhancing the auxin signal transduction process; genes encoding AUX/IAA (auxin/indole-3-acetic acid, Podel.08G185600 and Podel.05G230900) were up-regulated in ND vs. CK and MD vs. CD comparison groups, down-regulated in CD vs. ND, MD vs. ND, and FD vs. ND, indicating that drip irrigation and drip-fertilizer integration may be beneficial to AUX/IAA expression.

In the brassinosteroid (BR) signaling pathway, we found that the expression trends of BSK (BR-signaling kinase, Podel.01G257100) and CYCD3 (cyclin D3, Podel.14G022600 and Podel.07G056700) were consistent, both down-regulating expression in ND vs. CK and MD vs. CD, and up-regulating expression in CD vs. ND, MD vs. ND, FD vs. ND, and FD vs. MD, indicating that the addition of fertilizer and trace elements may be beneficial to poplar’s response to BR signaling.

In the Cytokinin (CTK) signaling pathway, the expression of AHP (histidine-containing phosphotransfer protein, Podel.13G028500), B-ARR (two-component response regulator ARR-B family, Podel.02G169600 and Podel.06G276700) is consistent in CD vs. ND, MD vs. ND, FD vs. ND, and FD vs. MD, with up-regulation, while down-regulated in ND vs. CK and MD vs. CD, indicating that the CTK signaling pathway may be positively regulated by fertilizer and trace elements.

### WGCNA analysis for mining hub genes associated with growth traits

**Fig. 8:**
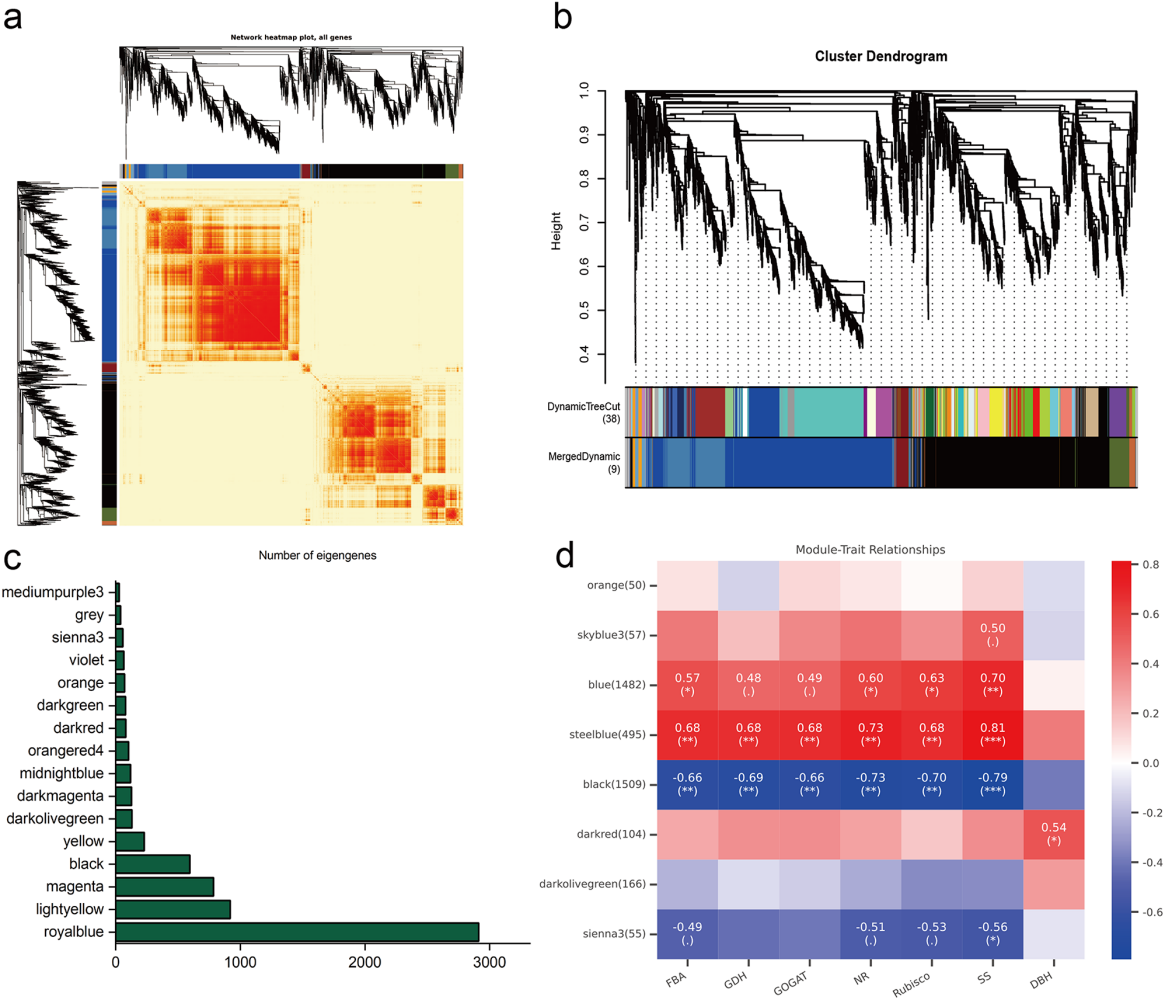
WGCNA analysis reveals modules associated with water and fertilization treatments. **a** Heatmap shows Pearson correlation between characterized genes in co-expressed gene modules. **b** Dendrogram for clustering different genes based on topological overlap with specified module colors. **c** Bar chart of the number of genes characterized in different modules. **d** Heatmap of the correlation between different modules and each indicator. Correlation coefficients are shown in different colors depending on the score

**Fig. 9:**
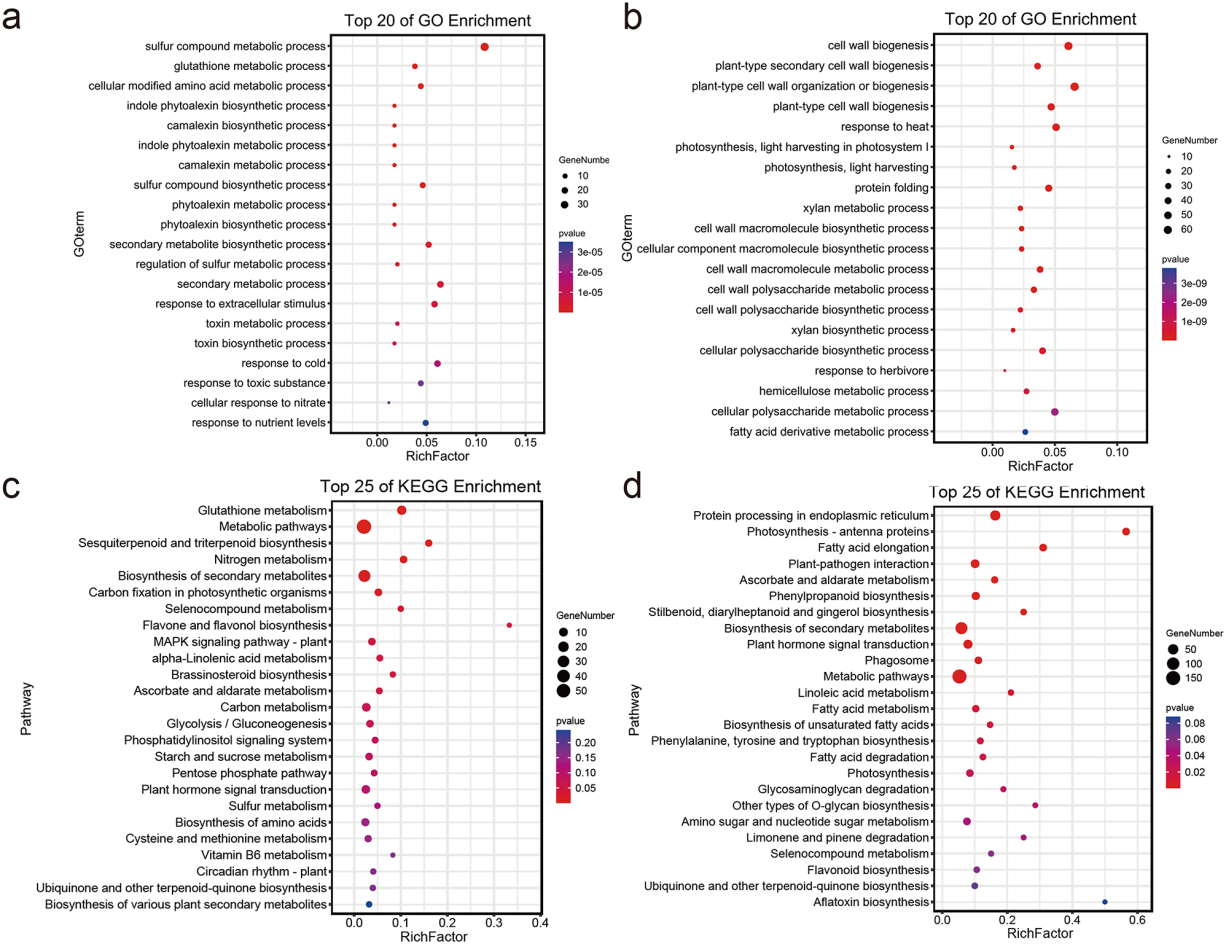
Biological processes of significant GO terms and KEGG pathways for eigengenes of different modules. **a** GO terms of the steelblue module. **b** GO terms of the black module. **c** KEGG pathways of the steelblue module. **d** KEGG pathways of the steelblue module. The x-axis represents the enrichment factor, and the y-axis represents the term/pathway. the size of each circle represents the number of genes, and the color represents the p-value, with a *P* ≤ 0.05 indicating significant enrichment

To mine genes related to growth and other important traits, we used a total of 29,762 genes expressed in poplar leaves under different water and fertilizer treatments, combined with physiological indicators such as growth, photosynthesis, and nitrogen metabolism-related enzyme activity, to perform WGCNA analysis [29] (Fig. 8). The median genes with standard deviation (SD) ≤ 0.05 were screened to eliminate genes with low expression variation, resulting in 3,984 genes. Highly related gene groups are categorized as a single module, and functionally equivalent genes typically exhibit the same expression trend when the module’s lower limit is set to 30 and the sensitivity of module formation to 26. Through pairwise correlation evaluation, these genes were divided into nine co-expression modules, with each highly correlated gene group corresponding to a branch of the tree (Fig. 8a and b). Within the same module, there is often a high degree of topological overlap between genes, and these modules can be clustered into two highly interconnected clusters. Among all modules, the three modules with the largest number of characteristic genes are black module (1509 genes), blue module (1482 genes) and steelblue module (495 genes), while the three modules with the fewest characteristic genes are orange module (50 genes), sienna3 module (55 genes) and skyblue3 module (57 genes) (Fig. 8c). To identify co-expression modules highly correlated with growth traits, Pearson correlation analysis was used to calculate the correlation coefficient and p-value between module feature genes and traits. Modules associated with FBA, GDH, GOGAT, NR, Rubisco activity, SS content, and DBH were screened using |*r*| ≥ 0.3 and *P* < 0.05 as thresholds. The steelblue module and black module were significantly positively correlated (0.68 < *r* < 0.81) and negatively correlated (-0.79 < *r* < -0.66), respectively, with the activity of FBA, GDH, GOGAT, NR, Rubisco, and SS content, while the darked module was only significantly positively correlated with DBH (*r* = 0.54) (Fig. 8d). Thus, with careful water and fertilizer management, it is hypothesized that the genes in the steelblue and black modules may be genes that down-regulate markers like photosynthesis and nitrogen metabolism enzyme activity. To explain the regulatory effects on growth traits for genes in the steelblue and black modules, further annotation was conducted using GO and KEGG enrichment analysis (Fig. 9). The enrichment results showed that the genes in the steelblue module mainly involved in the "sulfur compound metabolism" process, "glutathione metabolism", and cellular modified amino acid metabolic process, and participated in pathways such as "nitrogen metabolism" and "carbon fixation in photosynthetic organisms" (Fig. 9a and c). The majority of the black module’s genes are involved in "photosynthesis", "cell wall biogenesis", "light harvesting in photosystem I", "carbohydrate biosynthesis", and pathways like "photosynthesis - antenna proteins" (Fig. 9b and d). The transcription factor (TF) annotation results showed that the characterized genes in the steelblue and black modules included 256 and 736 TFs, respectively, which accounted for 49.50% of the total number of characterized genes and involved 53 gene families (Table S6). The most abundant TF families in the steelblue module are Dof and Whirly, and in the black module are bHLH. According to previous reports, multiple homologs of these TFs are involved in some processes such as carbon and nitrogen metabolism, growth and development, and response to adversity in plants [30–32]. Therefore, we postulated that precision water and fertilizer management might regulate poplar growth by regulating the expression of related genes involved in processes such as carbon and nitrogen metabolism. This, in turn, could lead to alterations in the activity of enzymes related to photosynthesis and nitrogen metabolism.

**Fig. 10:**
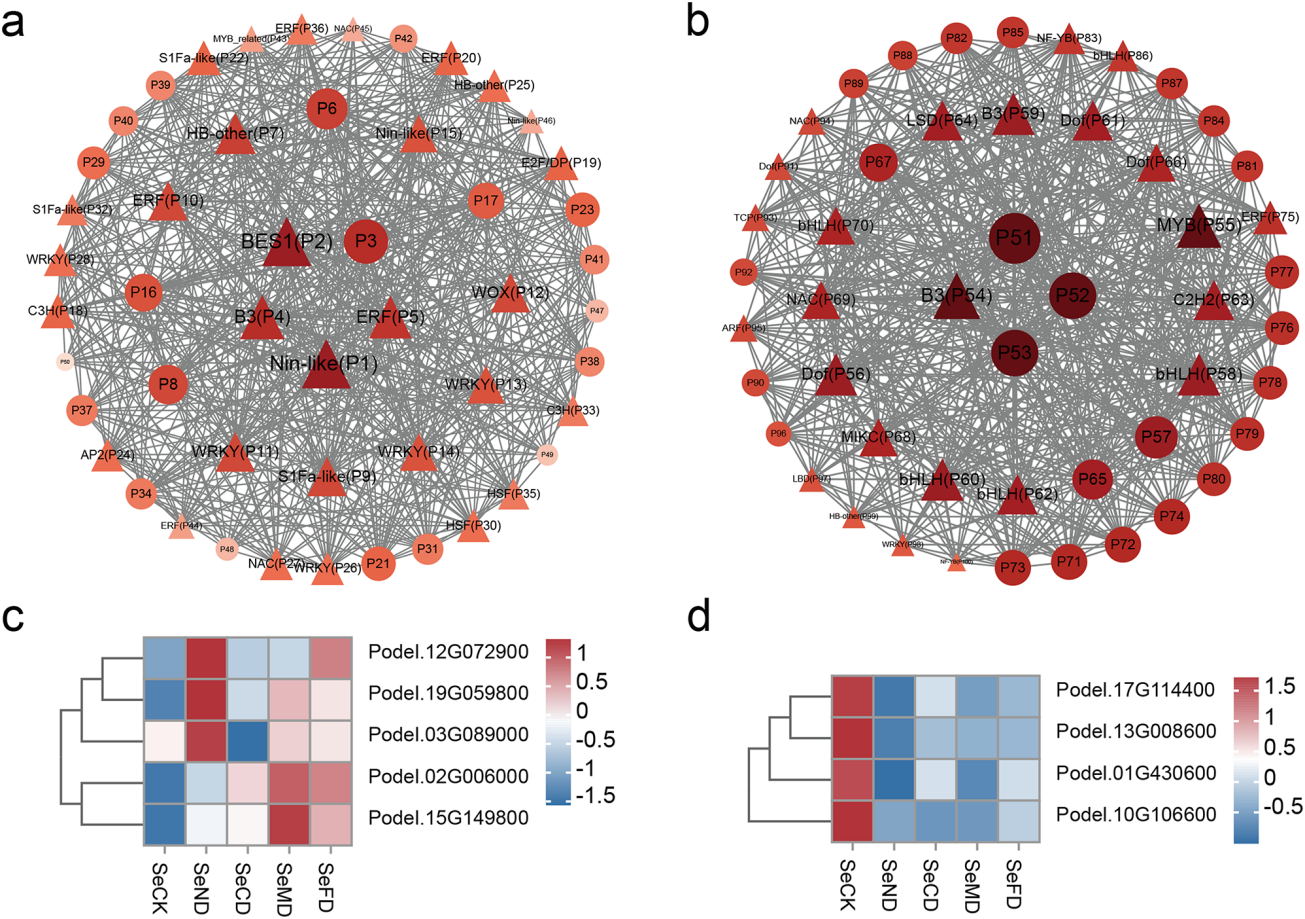
Co-expression networks and expression heatmap of hub genes. **a** steelblue module network. **b** black module network. **c** Heatmap of hub genes expression in steelblue module. **d** Heatmap of hub gene expression in the black module. The darker the node color, the higher the connectivity between the genes in the module. Triangles indicate transcription factors, and circles indicate structural genes

To further explore the genes for precision water and fertilizer regulation, the co-expression network was constructed for the genes with the top 50 weights in the steelblue and black modules. A total of 613 pairs of linear pairs were obtained (Table S7), and imported into Cytoscape 3.9.0 software for visualization (Fig. 10, Table S8). Five and four genes exhibiting the highest connectivity were chosen as hub genes, respectively (Fig. 10a.b). The hub genes within the steelblue module displayed up-regulation in response to both drip irrigation and water-fertilizer coupling. During water and fertilizer coupling treatment, gene expression was mainly down-regulated (Podel.12G072900, Podel.03G089000, and Podel.19G059800 were down-regulated, while Podel.02G006000 and Podel.15G149800 were up-regulated). Under the addition of trace elements, gene expression was mainly down-regulated (Podel.03G089000, Podel.02G006000, Podel.19G059800, and Podel.15G149800 were all down-regulated) (Fig. 10c). The hub genes of the black module were all down-regulated by drip irrigation (Podel.13G008600, Podel.01G430600, and Podel.10G106600 were significantly downregulated), and were up-regulated when trace elements were added. The gene expression trend is similar during water and fertilizer coupling, mostly up-regulated, while the expression trend is exactly opposite under water and fertilizer integration (Podel.17G114400, Podel.13G008600, and Podel.01G430600 are up-regulated in water and fertilizer coupling, and down-regulated in water and fertilizer integration). Functional annotation results indicated that these hub genes included Podel.12G072900 (ABC transporter A family member 7, Nin-like), Podel.03G089000 (Beta-amylase 3, BES1), Podel.02G006000 (Monosaccharide-sensing protein 2), Podel.19G059800 (Taxadiene 5-alpha hydroxylase, B3), Podel.15G149800 (Protein DETOXIFICATION 35, ERF). Podel.17G114400 (Protein GLUTAMINE DUMPER 3), Podel.13G008600 (Protein of unknown function, DUF538), Podel.01G430600 (COP1-interacting protein 7) and Podel.10G106600 (Plasmodesmata-located protein 6, B3) (Table 2), which are hypothesized to be closely related to nutrient uptake and translocation in poplar.

**Table 2:** Functional annotation of hub genes in the steelblue and black modules

Modules	Gene id	Arabidopsis Orthologs	Description	TF(Reference Species)	Orthologs
Steelblue	Podel.12G072900	AT3G47780	ABC transporter A family member 7	Nin-like (*Malus domestica*)	MDP0000265619
	Podel.03G089000	AT4G17090	Beta-amylase 3	BES1 (*Manihot esculenta*)	cassava4.1_002728m
	Podel.02G006000	AT4G35300	Monosaccharide-sensing protein 2	-	-
	Podel.19G059800	AT5G36110	Taxadiene 5-alpha hydroxylase	B3 (*Theobroma cacao*)	Thecc1EG000515t1
	Podel.15G149800	AT4G25640	Protein DETOXIFICATION 35	ERF (*Triticum urartu*)	EMS59827
Black	Podel.17G114400	AT4G25760	Protein GLUTAMINE DUMPER 3	-	-
	Podel.13G008600	AT1G56580	Protein of unknown function, DUF538	-	-
	Podel.01G430600	AT4G27430	COP1-interacting protein 7	-	-
	Podel.10G106600	AT2G01660	Plasmodesmata-located protein 6	B3 (*Malus domestica*)	MDP0000685403

### qRT-PCR validation

**Fig. 11:**
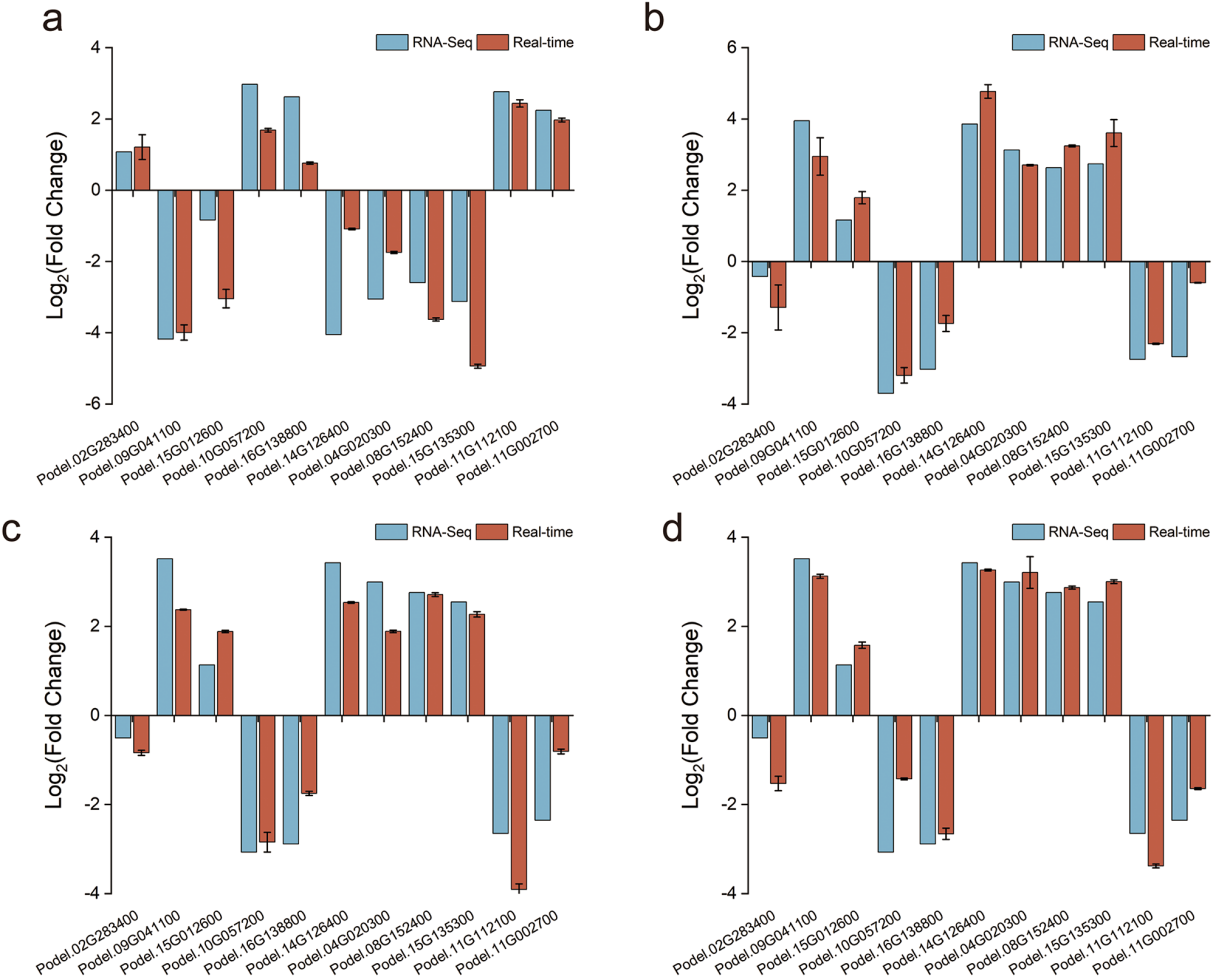
Quantitative verification of eleven genes in four comparison groups. The x-axis represents 11 randomly selected genes, and the y-axis represents the log_2_ Fold change value. **a**, **b**, **c**, and **d** indicate qRT-PCR validation results for genes in the ND vs. CK, CD vs. ND, M1D vs. ND, and F1D vs. ND comparison groups, respectively

To verify the accuracy of the RNA-seq results, we randomly selected 11 genes for qRT-PCR to detect the expression levels of genes in poplar leaves, and the results showed that the expression trends of these 11 genes were consistent with the results of RNA sequencing, indicating that transcriptome sequencing data were reliable in this study (Fig. 11).

## Discussion

### Precision water and fertilizer-intensive management regulates the physiological characteristics of poplar

It has been shown that proper nitrogen fertilization can increase N uptake and utilization efficiency, by enhancing the activities of enzymes related to N metabolism, such as NR, NiR, GS, and GOGAT, and promote crop growth [33]. After 3 months of continuous observation of physiological indicators of poplar plantation, such as Rubisco, FBA, NR, GDH, and GOGAT activities, as well as SS and Chl contents of leaf tissues, under different precision water and fertilizer combines of intensive management. We found that drip irrigation has no significant impact on various physiological indicators, compared with conventional irrigation. However, the combined water and fertilizer can effectively promote the activities of photosynthesis- and nitrogen metabolism-related enzymes, especially under precision drip irrigation and fertilizer management in September. These results suggest that appropriate water and nutrient management can promote the photosynthesis and nitrogen metabolism of poplar, which are beneficial to promote the growth and productivity of poplar plantations. Yin et al. [34] found that fertilization could increase the chlorophyll a/b content, intrinsic efficiency of photosystem II (Fv/Fm), and net photosynthetic rate of poplar leaves, which confirmed that fertilization can affect the related characteristics of photosynthesis and nitrogen metabolism in plants. In addition, we found that the chlorophyll content of poplar leaves mainly increased in the middle and late stages of fertilization (mainly in August and September), particularly in the water-fertilizer integration management containing trace elements increased significantly. Therefore, we hypothesized that the inclusion of Fe, Mn, and Zn in the fertilizer could enhance chlorophyll synthesis, a hypothesis supported by findings in other crops such as cabbage (*Brassica oleracea var. capitata*) and soybeans [35, 36].

### Expression pattern and functional analysis of DEGs under precision water and fertilizer-intensive management

The analysis of gene expression patterns under different precision water and fertilizer-intensive management showed that drip irrigation mainly has a negative regulatory effect on gene expression of poplar plantation, which was mainly related to cell wall synthesis, compared with conventional irrigation. This may be because the regimes of drip irrigation in this study are developed based on soil moisture content as an indicator, with more precision water requirements, which could reduce unnecessary water transport activities by down-regulating gene expression of cell wall synthesis [14, 15, 37]. Compared with drip irrigation without fertilizer, the gene expression pattern under different water-fertilizer combination management was consistent, which is mainly up-regulation. N, P, and K are the main elements added to fertilizers, which play an important role in promoting plant growth and productivity [38]. Thus we consider that nutrient addition may be the main reason for positive regulated gene expression in our study. Especially, up-regulated the genes involved in nitrogen metabolism and carbohydrate metabolism pathways. Studies showed that the low nitrogen supply could decrease the expression of photosystem I (PSI) and photosystem II (PSII) genes [39], and DEGs related to photosynthetic biotic nitrogen metabolism, carbon fixation, photosynthesis, starch, and sucrose metabolism, and zeatin synthesis were up- or down-regulated in rice under the appropriate nutrient conditions [21], indicating that water-fertilizer combination management contributes to improving nutrient uptake and translocation in poplar. In addition, a comparison of gene expression patterns under different water-fertilizer combination management (water-soluble fertilizer, MD and controlled-release fertilizer, CD) revealed that water-fertilizer integration may negatively affect gene expression, such as down-regulated energy supply-related genes. The amount of fertilizer applied each time may be different from water-soluble fertilizer and controlled-release fertilizer. Meanwhile, the application of trace elements (FD) up-regulated the expression of genes participating in secondary metabolic processes and secondary metabolite synthesis, similar to a study on thyme (*Thymus Vulgaris* L.) that, Fe foliar application increased the yield of secondary metabolites such as thymol oil and p-cymene [40].

### Precision water and fertilizer-intensive management regulates the expression of DEGs in different metabolic pathways

#### Photosynthesis

Nitrogen (N) is an important macronutrient that promotes plant carbon metabolism and growth, playing a crucial role in photosynthesis [35]. In this study, 15 photosynthesis genes were identified. Among these 5 photosystem I/II and photosynthetic electron transport genes (*PsbC*, *PsbR*, *Psb28*, *PsbN*, and *PsbF*), were up-regulated in comparison to ND vs. CK and MD vs. CD, suggests that drip irrigation and water-fertilizer integration management drive photosynthesis and electron transfer reactions of poplar. Nine DEGs encoding light-harvesting complex I/II chlorophyll a/b binding proteins (LHCA/B), which are membrane proteins in plant photosystem I, play a crucial role in the capture and transfer of energy during photosynthesis [41], up-regulated under drip-fertilization and drip-fertilization with trace elements application. In addition, the defects in Lhcb1/2 and Lhcb6, encoding peripheral light-trapping antennae, reduced light absorption [42] and inhibited the rate of electron transfer of *Arabidopsis* [43]. Thus, the application of nutrients and trace elements in drip irrigation can improve the photosynthesis ability of poplar by enhancing the capacities of light capture, light transport, and electron transport. This is confirmed by the significant increase in the activity of Rubisco and FBA, the key enzymes of photosynthesis, in precision water and fertilizer-intensive management.

### Nitrogen metabolism

The uptake, transport, assimilation, and redistribution of N are influenced by multiple genes and environmental factors. NRT can effectively regulate and transport NO_3_^−^, improving nitrogen utilization efficiency [44]. Three NRT genes were up-regulated under drip irrigation alone and water-fertilizer integration (Fig. S2), we hypothesized that accurate water supply could effectively regulate the nitrate uptake of poplar. Nitrate is converted to Glutamine and Glutamate during primary assimilation after being absorbed in plants, which are used to synthesize other amino acids and nitrogenous compounds [45]. GS and NR are two key nitrogen-assimilating enzymes, known as important players in the primary assimilation of nitrate and ammonium nitrogen of plants [46, 47]. One NR and one GS gene were up-regulated under drip-fertilization and drip-fertilization with trace elements management. This suggests that nutrients and trace elements play important roles in enhancing the nitrogen assimilation process of poplar. These results were consistent with the results of the activities determined by key enzymes of nitrogen metabolism, and the activities of NR, GDH, and GOGAT were significantly enhanced under drip-fertilizer combined.

#### Plant hormones

Plant hormones are considered important endogenous molecules that regulate plant growth, development, and tolerance to various stresses [22]. Indole-3-acetic acid (IAA) as an auxin, regulates plant development by inducing rapid cellular responses and changes in gene expression [48]. After auxin acts on plants, early auxin-responsive gene families such as Aux/IAA, GH3, and SAUR were rapidly induced and their gene expression was up-regulated, leading to ubiquitination and degradation of Aux/IAA, and activated ARF protein binding to exogenous promoter elements promotes growth hormone-responsive genes normalization [49–51]. Five auxin signal transduction-related genes were identified and all up-regulated under drip irrigation or drip-fertilizer combine management. Besides that, two *Aux/IAA* genes were significantly up-regulated in drip-fertilizer integration compared to CD, and one *ARF*, *GH3*, and *SAUR* genes were significantly up-regulated under drip-fertilizer with trace element integration compared to drip-fertilizer integration. Studies reported that nitrogen-containing compounds are key factors in the IAA signaling transduction, and N acts as a signaling molecule inducing the production of growth hormone [52]. BR *BSK* and *CYCD* are key genes in BR signaling. *BSK3* plays a bridging role in the BR signaling cascade from receptors to downstream genes, and it could enhance BR sensitivity and signaling to increase the extent of root under mild N deficiency [53–55]. CYCD3 as a key target of cytokinin in its regulation of the cell cycle, could promote cell division and be activated by BR signal [56, 57]. AHPs, and the paired response regulators (ARRs) core consist of a phosphorelay system in cytokinin (CTK) signaling transduction, after CTK perception, Phosphorylated AHPs phosphorylate type A or type B ARRs to initiate the expression of CTK-responsive genes [58–60]. One *BSK*, two *CYCD3*, one *AHP*, and two *B-ARR* genes were all up-regulated under different drip-fertilizer combines compared to drip irrigation. Above all, we conjecture that the drip-fertilizer combination or integration management benefited to interaction between plant hormones (IAA, BR, CTK) signal and nitrogen signal [61], and activated plant hormones signal transduction to regulate poplar growth.

### Analysis of the molecular mechanism of precision water and fertilizer-intensive management in regulating the growth of poplar

TFs are crucial regulators in controlling plant metabolism, growth, and development, which can coordinate nitrogen assimilation, carbon fixation, and growth of plants, such as rice [62–65]. In this study, two modules with significant positive (steelblue) or negative (black) correlations to carbon and nitrogen metabolism indicators were identified, including 992 genes, 49.5% of which were predicted as TFs, involving a total of 53 gene families. Co-expression network reveals nine hub genes were significantly up- or down-regulated expression under different drip fertilizer combines management (Fig. 10). Five out of them were predicted as TFs (Nin-like protein (NLP), BRI1-EMS-SUPPRESSOR1 (BES1), B3, and ethylene-responsive element binding factor (ERF)). It is reported that NLP7 as a transcription activator and an intracellular nitrate sensor, is involved in coordinate transport, hormone signaling transduction, and root-shoot development [66]. BES1 TF is a key mediator, connected to the BR signaling under nitrate-deficient by regulating the NRT in plants [67]. B3 proteins, constituting one of the largest TF families in plants, encompass families such as LAV, RAV, ARF, and REM. These proteins are primarily associated with signaling pathways related to hormones, including auxin, ABA, and brassinosteroids [68]. AP2/ERF TFs play important roles in biological and physiological processes such as hormone signaling transduction, metabolite regulation, and stress response [69].

**Fig. 12:**
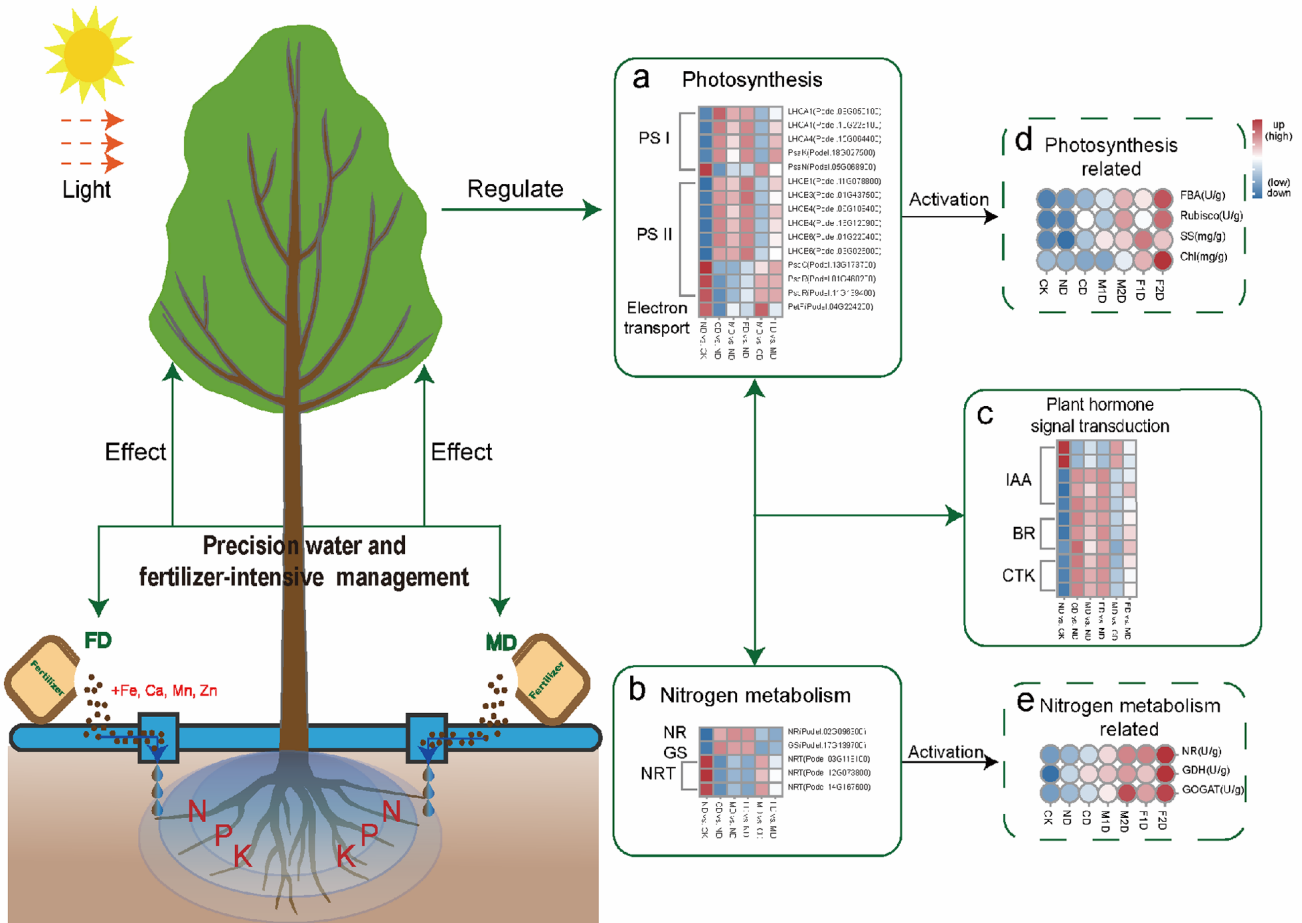
Schematic model of poplar under precision water and fertilizer-intensive management. Important DEGs in *Populus × euramericana* 'Neva' under precision water and fertilizer-intensive management are involved in nitrogen metabolism, photosynthesis, and plant hormone transduction. The circles of different colors represent the size of the measured physiological and biochemical indicators. The bluer the color, the smaller the value, the redder the color, and the larger the value

Therefore, we consider that precision drip fertilizer combined management could regulate the growth by activating the coordinated regulatory role of carbon and nitrogen metabolism (Fig. 12). After precision water and fertilizer is applied, it can activate the expression of NRT-related genes to promote nitrate transport, and then regulate the expression of N-assimilation enzymes such as NR and GS (Fig. 12b) to increase the activity of NR, GDH, and GOGAT enzymes (increased by 1.28–2.63 times compared with drip irrigation) (Fig. 12e), as well as enhance the process of nitrogen assimilation process to promote nitrogen absorption and utilization finally. Besides, after absorption, nitrate can activate TFs such as nitrate sensors, to activate plant hormone signal transduction (BR, IAA, and CTK), which is beneficial to regulate the growth of poplars (Fig. 12c). In addition, amino acids converted from nitrogen assimilation were transported to organs such as leaves by transport proteins, which supporting the process of photosynthetic carbon assimilation. These factors could up-regulate the expression of several key photosynthesis genes (*Psbs*, *LHCs*, *Ftsh1*, *Ftsh8*), and increase the key enzyme activity of Rubisco (increased by 1.05–2.68 and 1.46–2.56 times compared with CK and ND, respectively) and FBA (increased by 1.11–2.09 and 1.12–1.89 times compared with CK and ND, respectively) enhancing the capabilities of light capture, light transport, and electron transport for photosynthesis. Ultimately, promoted the accumulation of photosynthetic products such as soluble sugars (increased by 0.96–1.34 and 1.12–1.89 times compared with CK and ND, respectively), thus accelerating poplar growth.

Under the precision water and fertilizer-intensive management, the alterations in physiological and biochemical indicators and gene expression have revealed the response of poplar’s nitrogen metabolism and photosynthesis to specific water and fertilizer management strategies, offering a scientific foundation for understanding the impact of these management practices on the physiological and molecular levels of poplars; In conjunction with phenotype and gene expression data, this research delves into the relationships between gene expression and key traits affecting yield in plantations. It identifies key genes influencing yield-related traits, providing a scientific rationale and reference for developing effective and precision water and fertilizer management strategies, optimizing the expression of target genes, and offering theoretical guidance for enhancing forest production and management, as well as advancing forestry technology innovation capabilities and standards.

## Conclusion

Precision drip and fertilizer combined management significantly affects the differential expression of numerous genes, the key enzyme activity of nitrogen assimilation and photosynthesis, as well as the accumulation of photosynthetic products in the leaves of *Populus × euramericana* plantation. In this study, precision water and fertilizer combined management could regulate the expression of core genes and TFs in multiple biological processes such as carbon and nitrogen metabolism, and plant hormone signal transduction, lead an increase in the activity of key enzymes in related processes and enhanced nitrogen absorption and utilization, and photosynthesis capacity, at last collaborative regulate the growth of poplar. Co-expression network identified nine hub genes regulated by precision drip and fertilizer combined management, which may play a crucial role in regulating the growth of poplar. These findings serve as a foundational reference for advocating highly efficient, precision intensive management to achieve the optimal expression of the target genes.

### Electronic supplementary material

Below is the link to the electronic supplementary material.

Supplementary Material 1

Supplementary Material 2

## Data Availability

The sequenced clean reads generated in this study have been deposited in the Genome Sequence Archive [70] in National Genomics Data Center [71], China National Center for Bioinformation / Beijing Institute of Genomics, Chinese Academy of Sciences (GSA: CRA015045) that are publicly accessible at https://ngdc.cncb.ac.cn/gsa.

## Abbreviations

DEG: Differential expression gene
KEGG: Kyoto Encyclopedia of Genes and Genomes
GO: Gene Ontology
FPKM: Fragments Per Kilobase Millon Mapped Reads
Rubisco: Ribulose-1,5-bisphosphate carboxylase/oxygenase
FBA: Fructose 1,6 bisphosphate aldolase
NRT: Nitrate transporters
NR: Nitrate reductase
GS: Glutamine synthetase
GDH: Glutamate Dehydrogenase
GOGAT: Glutamate synthase
SS: Soluble sugar
Chl: Chlorophyll
AUX/IAA: Auxin-responsive protein IAA
CTK: Cytokinin
BR: Brassinosteroid
