# Supplementary material for: RNA-seq reveals the gene expression in patterns in Populus × euramericana 'Neva' plantation under different precision water and fertilizer-intensive management
Source: BMC Plant Biol. 2024 Aug 9;24:759. doi: 10.1186/s12870-024-05427-4 (PMC11312740; doi:10.1186/s12870-024-05427-4)
Supplement: Supplementary file 1 — Supplementary Material 1 [file 12870_2024_5427_MOESM1_ESM.docx]

Additional file Fig. S1 Correlation heat map between different samples.

Additional file Fig. S2 Heatmap representing the expression levels of NRT, NR, and GS genes in CK, ND, CD, MD, and FD. Expression level based on FPKM data.
